# *TREM2* is associated with tumor immunity and implies poor prognosis in glioma

**DOI:** 10.3389/fimmu.2022.1089266

**Published:** 2023-01-11

**Authors:** Mingchen Yu, Yuanhao Chang, You Zhai, Bo Pang, Peng Wang, Guanzhang Li, Tao Jiang, Fan Zeng

**Affiliations:** ^1^ Department of Molecular Neuropathology, Beijing Neurosurgical Institute, Capital Medical University, Beijing, China; ^2^ Department of Neurosurgery, Beijing Tiantan Hospital, Capital Medical University, Beijing, China; ^3^ China National Clinical Research Center for Neurological Diseases, Beijing, China; ^4^ Chinese Glioma Genome Atlas Network (CGGA) and Asian Glioma Genome Atlas Network (AGGA), Beijing, China; ^5^ Research Unit of Accurate Diagnosis, Treatment, and Translational Medicine of Brain Tumors, Chinese Academy of Medical Sciences, Beijing, China

**Keywords:** glioma, TREM2, macrophage, prognosis, tumor immunity

## Abstract

Triggering receptor expressed on myeloid cells 2 (TREM2) is expressed in myeloid cells of the central nervous system (CNS), which mediate the immunological response in a variety of diseases. Uncertain is the function of TREM2 in glioma and tumor immune responses. In this research, the expression patterns of TREM2 in glioma were analyzed, along with its prognostic value and functional roles. TREM2 expression is increased in glioblastomas, gliomas with a mesenchymal subtype, gliomas with wild-type isocitrate dehydrogenase, and gliomas without 1p/19q deletion, all of which suggest the aggressiveness and poor prognosis of gliomas. Gene ontology, KEGG, and Gene set variation analyses indicated that TREM2 may serve as an immune response mediator. However, the function of T cells against tumor cells was negatively correlated with TREM2, suggesting that TREM2 may suppress tumor immunity. Further investigation demonstrated a correlation between TREM2 expression and immune checkpoint expression. CIBERSORT research revealed a link between a higher TREM2 expression level and the enrichment of tumor-associated macrophages, especially M2 subtype. Single-cell analysis and multiple immunohistochemical staining results showed that microglia and macrophage cells expressed TREM2. Immunofluorescent staining indicated that knocking down the expression of TREM2 would result in a decrease in M2 polarization. TREM2 was discovered to be an independent prognostic factor in glioma. In conclusion, our findings revealed that TREM2 was significantly expressed in microglia and macrophage cells and was intimately associated with the tumor immune microenvironment. Thus, it is expected that small-molecule medications targeting TREM2 or monoclonal antibodies would enhance the efficacy of glioma immunotherapy.

## 1 Introduction

Glioma is the most prevalent primary brain tumor and is notorious for its aggressiveness, recurrence, and poor prognosis (1). It constitutes 81% of malignancies of the central nervous system, with a median survival duration of fewer than 15 months and a 5-year survival rate of 10% (2). In recent decades, glioma treatment has progressed from basic surgical resection to personalized treatment, involving safe resection guided by a neuro-navigation system, postoperative radiation, and chemotherapy guided by molecular pathology (3, 4). Despite the modified treatment, glioma does have an unfavorable prognosis. Due to their contribution to malignancy, the tumor microenvironment and noncancerous cells that are recruited into the tumor microenvironment have garnered growing interest (5). Immune cells are of particular concern owing to their dominance as glioma components (6). Immunotherapy is a novel treatment approach with promising potential applications. Recent successes of immune checkpoint inhibitors and chimeric antigen receptor T cell therapy in the treatment of other solid tumors demonstrate the potential of immunotherapy in the treatment of glioblastoma (7, 8). Concurrently, efforts have been made to identify the immunological features of gliomas to develop novel immunotherapeutic strategies (9). It will be effective to enhance the prognosis of glioma patients by searching for novel immunotherapeutic targets.

TREM2 is a transmembrane receptor in the immunoglobulin superfamily and a major pathologically induced immune signaling center (10). It consists of an extracellular domain with a V-type immunoglobulin domain and a short ligand peptide, a single transmembrane helix, and a short intracellular domain without a signal transduction motif (11). TREM2 is widely expressed on the surface of the microglia cell membranes, and its ligands contain various free and bound anionic molecules, bacterial products, DNA, lipoproteins, and phospholipids (12, 13). After binding to the ligand, TREM2 transmits intracellular signals through the DNAX-activating protein (DAP12) (14). DAP12, also known as TYRO protein tyrosine kinase binding protein, is a signal transduction connector protein expressed predominantly in microglia and other cells involved in innate immune responses (15). DAP12 is phosphorylated on tyrosine by protein kinase SRC, which recruits the tyrosine-protein kinase SYK, and that in turn recruits various signaling mediators and adapters, resulting in intracellular signal transmission (16).

In physiological states, TREM2 signals from various environments induce significant changes in cell phenotype and biological function, including induction of phagocytosis, lipid metabolism and metabolic metastasis, promotion of cell survival, and anti-inflammatory activation (17). Previous studies have shown that TREM2 coding variations enhance the risk of Alzheimer’s disease and other neurodegenerative diseases (18). In addition, an increasing number of research show that TREM2 plays a role in tumor progress (19, 20). TREM2 is expressed in cancer cells and directly promotes survival in esophageal cancer, according to studies (21). Moreover, some researchers found that TREM2 is highly expressed in TAMs in non-small cell lung cancer and liver cancer patients (22, 23), suggesting that the high expression of TREM2 is associated with poor prognosis of tumor patients. However, the role of TREM2 in glioma and tumor immune responses remains unclear.

In this study, we systematically investigated TREM2 expression and its role in glioma prognosis using clinical samples and RNA sequencing data from the Cancer Genome Atlas (TCGA) and the Chinses Glioma Genome Atlas (CGGA), focusing on the molecular characteristics, immunological characteristics, and prognostic significance of TREM2. We also evaluated the correlation between TREM2 expression and key immune-related components. A series of bioinformatic analysis methods, including gene ontology analysis, KEGG analysis, gene set variation analysis, and CIBERSORT, were employed to find out the role of TREM2 in the glioma immune process in our study. Immunostaining was performed to confirm the expression pattern of TREM2 and its correlation with M2 subtype macrophages, suggesting that TREM2 was intimately related to the tumor immune microenvironment. These results indicated that TREM2 was a potential independent prognostic factor and immunotherapeutic target, which might provide novel insights into the treatment of glioma.

## 2 Results

### 2.1 High expression of TREM2 was associated with glioma malignancy

**Figure 1:**
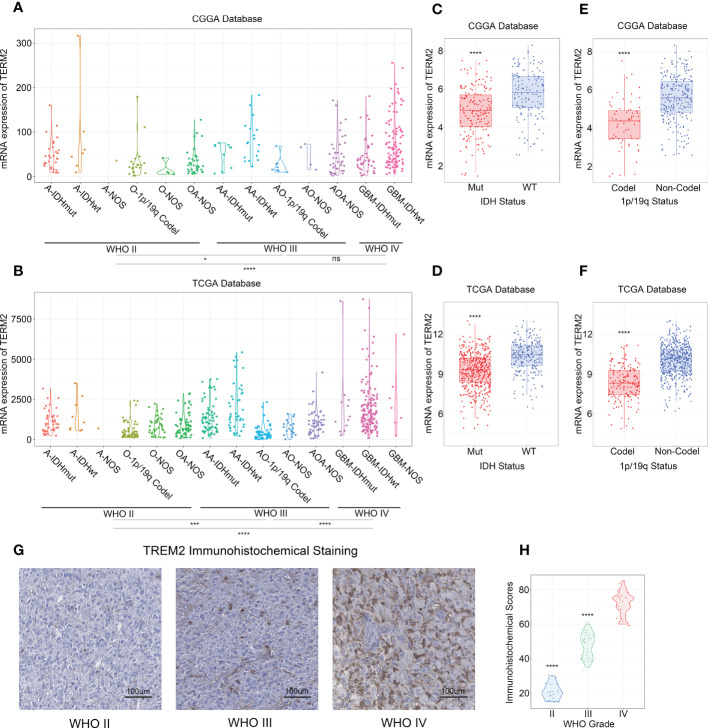
High expression of TREM2 was associated with malignant glioma. **(A, B)** TREM2 was significantly increased in GBM (WHO grade IV) in CGGA and TCGA databases. **(C, D)** TREM2 was significantly increased in IDH wildtype gliomas in CGGA and TCGA databases (Mut: IDH mutation; WT: IDH wildtype). **(E, F)** TREM2 was significantly increased in 1p/19q non-codeletion gliomas in CGGA and TCGA databases (Codel: 1p/19q codeletion; Non-Codel: 1p/19q non-codeletion). **(G)** The representative photos of IHC staining of TREM2 in different glioma grades. **(H)** The Immunohistochemical Scores of TREM2 were measured in different grades. 19, 30 and 25 patients were from Grade II, Grade III and Grade IV, respectively. A, Astrocytoma; O, Oligodendroglioma; OA, Oligoastrocytoma; AA, Anaplastic astrocytoma; AO, Anaplastic oligodendroglioma; AOA, Anaplastic Oligoastrocytoma; GBM, Glioblastoma. ns, *, *** and **** indicate no statistical significance, p < 0.05 and p < 0.0001, respectively.

To investigate whether TREM2 was an oncogene, we compared the expression levels of TREM2 in 31 distinct tumors and their corresponding normal tissues using GEPIA (Gene Expression Profiling Interactive Analysis). The results showed that TREM2 was highly expressed in GBM and LGG tissues rather than normal tissues or any other tumor types, indicating that TREM2 may play a carcinogenic role in glioma (**Supplementary Figure 1**). To investigate the significance of TREM2 in glioma progression, we analyzed the RNA-seq data of bulk tumor tissues from patients with different molecular pathological backgrounds obtained from TCGA and CGGA databases. CGGA databases were divided into two independent cohorts, each including 325 and 693 patients. First, compared to WHO II and WHO III gliomas, we found that WHO IV GBM patients had relatively higher TREM2 expression in the three databases (**Figures 1A, B** and **Supplementary Figure 2A**), demonstrating that high TREM2 expression was predictive of high malignancy of glioma. Next, we explore the expression of TREM2 in glioma tissues through IHC staining. Consistent with the RNA-seq data, we found that TREM2 was enriched in GBM tissues (**Figures 1G, H**).

In addition, we investigated TREM2 expression independently in each of the three databases, with IDH mutation status and 1p/19q codeletion status considered. IDH mutation status was the classical biomarker for predicting prognosis in gliomas, and patients with IDH mutations typically had longer overall survival. Moreover, 1p/19q codeletion status was a signature to predict the radiotherapy sensitivity. Patients with low-grade oligodendroglioma who had the 1q/19q chromosomal codeletion benefited more from radiation. Patients with wild-type IDH or 1p/19q non-codeletion showed the highest TREM2 expression level across all databases (**Figures 1C–F**, and **Supplementary Figures 2B, C**). Furthermore, through evaluating the status of chromosome instability in the TCGA database, we discovered that the low expression level of TREM2 is usually accompanied by 1p/19q co-deletion, whereas the high expression level of TREM2 is typically accompanied by amplification of chromosome 7 and deletion of chromosome 10 (**Supplementary Figure 3**). These results indicated that the high expression of TREM2 predicted a high malignant glioma.

### 2.2 TREM2 was an indicator for mesenchymal glioma

**Figure 2:**
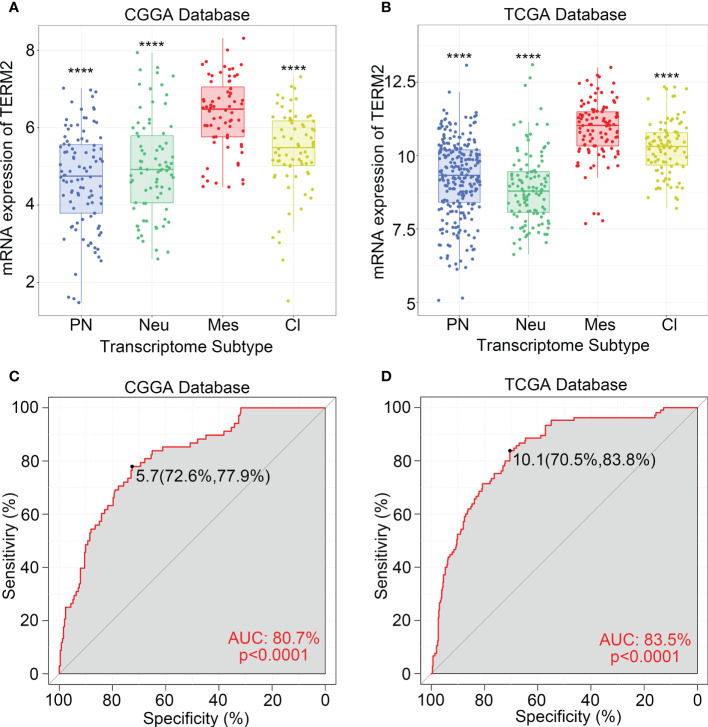
TREM2 was an indicator of mesenchymal glioma. **(A, B)** TREM2 was highly expressed in the Mesenchymal subtype in the CGGA database and TCGA database. **(C, D)** ROC curve analysis showed that TREM2 was high sensitivity and specificity to predict the Mesenchymal subtype in the CGGA database and TCGA database. Differences between groups were tested by Tukey’s multiple comparisons test. **** indicates p < 0.0001.

To investigate the molecular expression pattern of TREM2, we explored the distribution of TREM2 expression in different molecular subtypes of glioma, which were identified by the TCGA network based on transcriptomic and genomic dimensions. As a result, TREM2 was dramatically upregulated in the mesenchymal subtype compared to the other three subtypes, in CGGA, CGGA(2019) and TCGA databases (**Figures 2A, B** and **Supplementary Figure 2D**). To further validate this finding, ROC curves were used to evaluate the discrimination ability of TREM2 expression for mesenchymal subtype in all grades of glioma. The area under the curve (AUC) of TREM2 expression was surprisingly up to 80.7%, 76.9%, and 83.5% in CGGA, CGGA (2019) and TCGA databases, respectively (**Figures 2C, D** and **Supplementary Figure 2E**). According to these observations, TREM2 may serve as a biomarker for mesenchymal subtype glioma due to its highly specific expression in mesenchymal subtype gliomas.

### 2.3 TREM2 was correlated with immune functions in glioma

**Figure 3:**
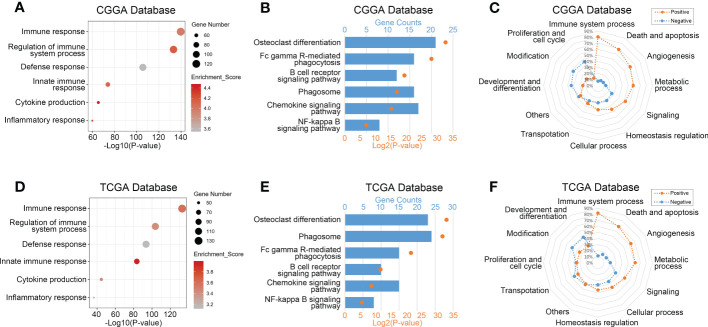
TREM2 was correlated with immune functions in glioma. **(A, D)** Gene ontology analysis showed that TREM2 was mostly associated with immune response, regulation of immune system process, defense response, and inflammatory response in both the CGGA database and TCGA database. **(B, E)** KEGG pathway analysis showed that TREM2 was mostly involved in immune response related pathway in both CGGA database and TCGA database. **(C, F)** TREM2 had positive correlation with 80.46% and 81.90% of biological functions of the immune system process in CGGA and TCGA databases, respectively.

To explore biological processes related to TREM2 expression in glioma, we performed Pearson’s correlation analysis to screen genes strongly positively correlated with TREM2 (Pearson R> 0.5, p<0.0001). 597, 430, and 786 genes were identified in CGGA, CGGA (2019) and TCGA databases, respectively. Then, we entered these gene lists into DAVID Bioinformatics Resources 6.8 to perform Gene Ontology and KEGG analysis. Finally, the gene function was sorted by p-value in increasing order. Positively correlated genes in three databases were primarily enriched in biological processes such as immunological response, defense response, innate immune response, cytokine generation, and inflammatory response, according to the results (**Figures 3A, D** and **Supplementary Figure 4A**). In addition, we further investigated the relevant signaling pathway of TREM2-related genes through KEGG analysis. Results in **Figures 3B, E** and **Supplementary Figure 4B** showed that related genes were strongly connected with immune response pathways, including FcγR-mediated phagocytosis, B cell receptor signaling pathway, phagosome, chemokine signaling pathway, and NF-κB signaling pathway. These results revealed that TREM2-related genes mainly participated in the immune response of glioma.

### 2.4 TREM2-related immune response

To validate further the significance of TREM2 in glioma immune response, we first drew a heatmap to illustrate the correlation between TREM2 and several genes involved in immune function. The results showed a distinct positive correlation between them. Then we created a landscape of TREM2 expression and corresponding clinical patient features (**Supplementary Figures 4D–F**). Interestingly, we discovered that a high TREM2 expression level indicated high immunological activity, but a poor prognosis for patients. These results suggested that TREM2 may play a complicated role in the immune response to glioma. Finally, we conducted GSVA to assess their relationship. Downloaded from the AmiGO2 web portal, 7334 identified biofunctions were categorized into 12 groups, including immune system process, death and apoptosis, angiogenesis, metabolic process, signaling, cellular process, homeostasis regulation, transportation, proliferation and cell cycle, modification, development and differentiation, and others. Pearson correlation analysis was performed to analyze the correlation between TREM2 and these GO terms. In CGGA, CGGA (2019) and TCGA databases, 80.46%, 80.87%, and 81.90% of biological functions of immune system processes were positively correlated with TREM2, respectively (**Figures 3C, F** and **Supplementary Figure 4C**). These results demonstrated conclusively that TREM2 was essential for the glioma immune response.

### 2.5 TREM2 was associated with T cell immune response and inhibitory immune checkpoints

**Figure 4:**
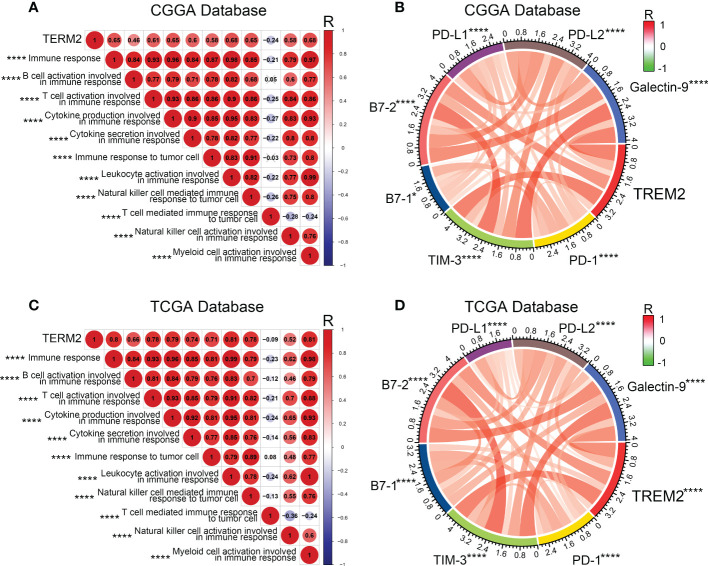
TREM2 was associated with T cell immune response and inhibitory immune checkpoints. **(A, C)** The correlation coefficient between TREM2 and immune function scores in glioma. The red circle represents a positive correlation. The blue circle represents a negative correlation. The grey “×” represents no significant correlation. Similar results have been found in both CGGA and TCGA databases. **(B, D)** The Pearson correlation coefficient between TREM2 and immune checkpoint expression in CGGA and TCGA databases. * and **** indicates p<0.05 and p < 0.0001, respectively.

Various immune cells, including T cells, B cells, NK cells, macrophages, dendritic cells, and others, mediated the immunological response. These immune components infiltrated the tumor microenvironment and either directly destroyed tumor cells or facilitated them in evading immunological surveillance. Considering the correlation between high TREM2 expression and poor prognosis, we hypothesized that TREM2 may enhance tumor immune evasion. To determine the precise function of TREM2, we analyzed the correlation coefficient between TREM2 and 11 types of immune system processes contained in three databases (**Figures 4A, C** and **Supplementary Figure 5A**). As anticipated, TREM2 was positively related with the majority of tumor immune functions, with the exception of “T cell-mediated immune response to tumor cell.” To avoid T cell-mediated immune response, tumor cells frequently upregulate immune checkpoint genes, such as PD-L1, PD-L2, and Galectin-9. Based on our findings, we hypothesized that TREM2 might modulate the expression of immunological checkpoints to assist glioma cells in maintaining malignant phenotypes. By investigating the correlation between TREM2 and a series of immune checkpoints in three databases, we validated that TREM2 was positively correlated with inhibitory immune checkpoints (**Figures 4B, D** and **Supplementary Figure 5B**). These results demonstrated that TREM2 may inhibit T cell-mediated immune response through immune checkpoints.

### 2.6 TREM2 was associated with tumor-associated macrophages

**Figure 5:**
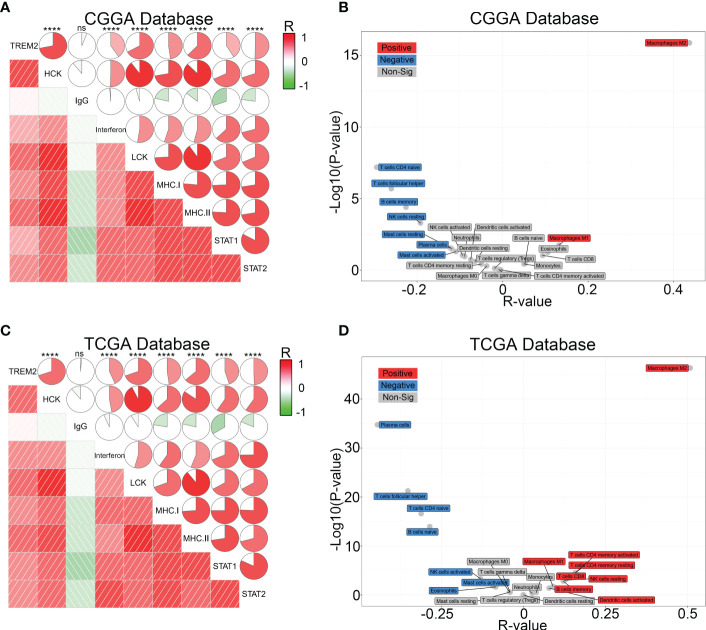
TREM2 was associated with inflammatory response and infiltrated immune cells. **(A, C)** The relationship between TREM2 and inflammatory activities in glioma. The correlation between TREM2 and other functions was analyzed by Pearson correlation analysis. ns, and **** indicate no statistical difference and p < 0.0001, respectively. **(B, D)** The relationship between TREM2 and infiltrated immune cells in both CGGA and TCGA databases. The red box represents a positive correlation and the blue box represents a negative correlation. The grey box represents no significance.

**Figure 6:**
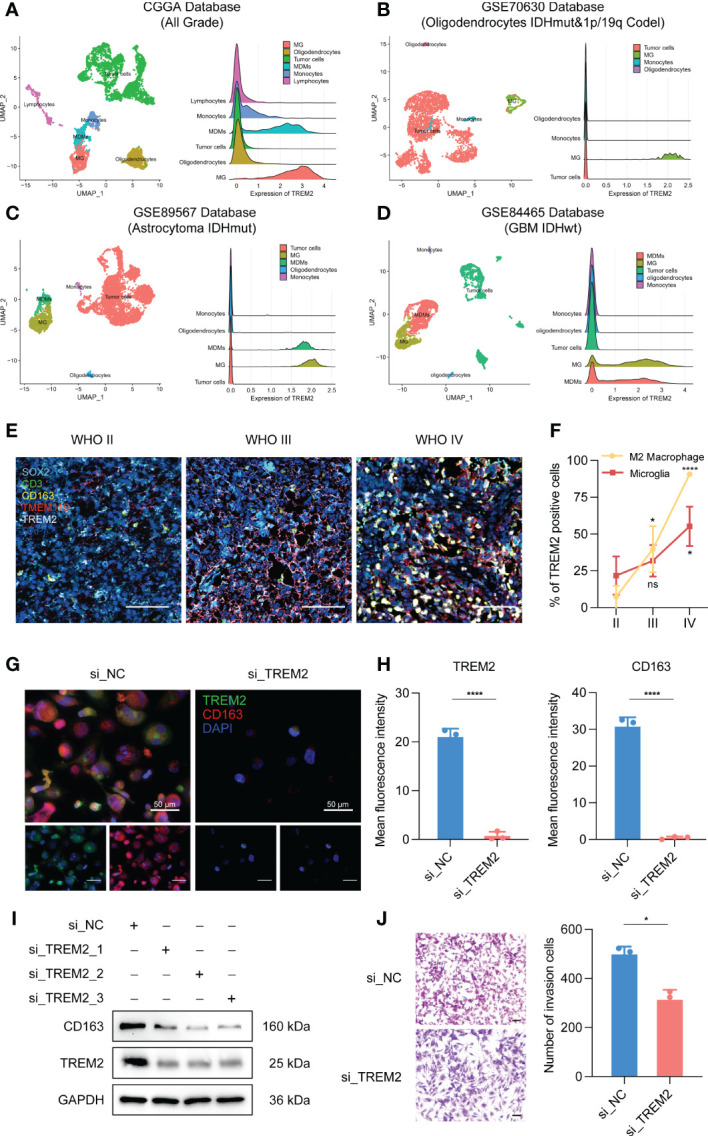
TREM2 was associated with tumor-associated macrophages. **(A–D)** The relationship between TREM2 and different immune cells in CGGA, GSE70630, GSE89567, and GSE84465 databases. The correlation between TREM2 and infiltrated immune cells was analyzed by Pearson correlation analysis. (MG: Microglia; MDMs: Macrophages) **(E)** The representative photos of polychromatic immunohistochemical staining of SOX2, CD3, CD163, TMEM119, TREM2, and DAPI in different grades of glioma tissue (aqua: SOX2; green: CD3; gold: CD163; red: TMEM119; white: TREM2; dark blue: cell nuclei). The scale bar is 100μm. **(F)** The bar chart shows the TREM positive cell proportion in microglia cells and M2 macrophage images. **(G)** The representative photos of IF staining of TREM2 in M2 macrophages. The left three photos are normal M2 macrophages. The right three photos are M2 macrophages which are polarized from si-TREM2 THP-1 cells. Green fluorescence is TREM2 and red fluorescence is CD163. Above is the merged photo of the below two. Cell nuclei are stained with DAPI. The scale bar is 50μm. **(H)** The bar chart shows the mean fluorescence intensity of TREM2 and CD163 positive cells in M2 macrophage IF images. **(I)** After knocking down TREM2 by siRNA, western blot assay was performed to detect the expression of TREM2 and CD163. **(J)** The representative images and bar chart of invasion transwell assay after co-culture. The scale bar is 100μm. ns, *, *** and **** indicate no statistical difference, p < 0.05, p<0.001 and p<0.0001, respectively.

To further investigate the influence of TREM2 in glioma immune response, we analyzed the correlation between TREM2 and inflammatory response. TREM2 was consistently positively correlated with the expression of HCK, interferons, MHC-I, MHC-II, STAT1, and STAT2, and negatively associated with the expression of IgG in three databases (**Figures 5A, C** and **Supplementary Figure 5C**). TREM2 was implicated in a variety of immune processes, including biofunctions mediated by T cells, NK cells, dendritic cells, neutrophils, and macrophages, but it inhibited B cell activation, according to these findings. To infer the fraction of immune and stromal cells of each patient, the immune score and stromal score were evaluated in CGGA and TCGA databases. Immune and stromal scores were found to be favorably correlated with the TREM2 expression (**Supplementary 6A, B, D, E**). In addition, gliomas with limited tumor purity demonstrated elevated TREM2 expression (**Supplementary Figures 6C, F**). These results revealed a significant correlation between the abundance of immune cells and TREM2 expression. Therefore, to determine which types of immune cells were tightly correlated with TREM2 in the tumor microenvironment, we performed a CIBERSORT analysis in three databases (**Figures 5B, D**, and **Supplementary Figure 5D**). The results revealed that M2 macrophage was dramatically correlated with TREM2 in three databases. Considering the substantial link between TREM2 and macrophages, we hypothesized that TREM2 might be expressed in tumor-infiltrated macrophages. To validate our hypothesis, we conducted single-cell sequencing analysis from glioma patients of different grades in CGGA, GSE70630, GSE89567, and GSE84465 databases (**Figures 6A-D**). The results showed that microglia cells and macrophages express TREM2. Moreover, polychromatic immunohistochemical staining demonstrated that TREM2 was co-localized with the surface markers of microglia and macrophages (**Figures 6E, F**). These findings suggested that TREM2 may remodel the microenvironment *via* macrophages. Immunofluorescent assay showed that polarized TREM2-knockdown THP-1 cells expressed less CD163 (M2-phenotype marker) compared to control group (**Figures 6G, H, I**). We found glioma cells that co-cultured with TREM2-knockdown THP-1 cells exhibited reduced invasive capacity compared with control group (**Figure 6J**). These results indicated that TREM2 reshaped the microenvironment through regulating macrophages polarization and altered the glioma invasiveness through TAMs.

### 2.7 TREM2 predicted worse survival in glioma

**Figure 7:**
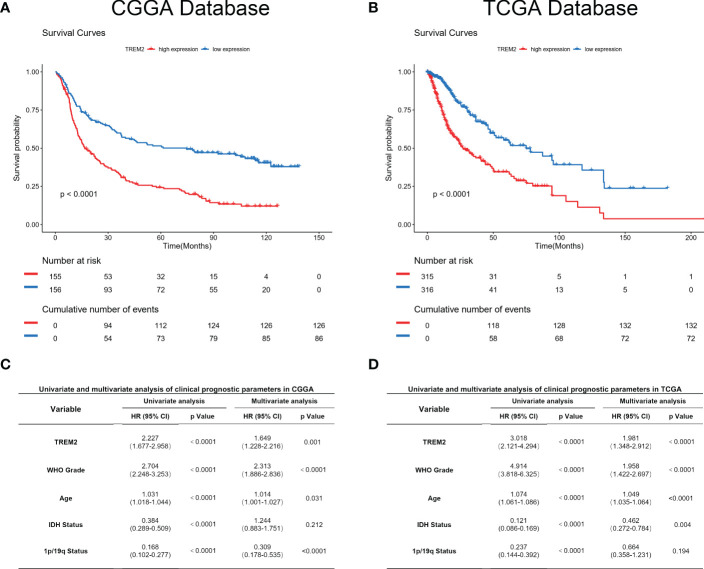
TREM2 predicted worse survival in glioma. **(A, B)** Clinical outcomes of patients with gliomas of low or high expression of TREM2. Kaplan-Meier survival analyses were performed in both CGGA and TCGA databases. **(C, D)** Univariate and Multivariate analyses of clinical prognostic parameters in both CGGA and TCGA databases.

As TREM2 played a pivotal role in the glioma immune response, we analyzed its potential for predicting patient prognosis. Kaplan-Meier curve was used to analyze the prognosis of all grades of glioma patients in CGGA and TCGA databases (**Figures 7A, B**). As expected, increased TREM2 expression was associated with shorter overall survival. This result indicated that TREM2 may be a malignant biomarker. Furthermore, Cox regression analysis was done on three databases to determine whether TREM2 was an independent clinical predictive factor. TREM2 expression, WHO Grade, age at diagnosis, IDH status, and 1p/19q co-deletion status were found to be significantly associated with glioma patients’ overall survival. Multivariate analysis further confirmed that the TREM2 expression was a significant predictor after adjusting for the aforementioned clinical factors (**Figures 7C, D**). These findings suggested that TREM2 may serve as an indicator of the poor prognosis in gliomas.

## 3 Discussion

Despite many efforts to reduce mortality, glioma remains the most aggressive intracranial tumor type in the central nervous system and has a severe impact on patient health (1, 2, 4). Over the past decades, radiotherapy and chemotherapy has been considered as the most effective treatment besides neurosurgical procedures (24). As the only clinical first-line chemotherapy drug, temozolomide has shown obvious resistance (25). Therefore, the prognosis for patients with glioma is still dismal. With the development of targeted therapy and immune therapy, a series of new treatments have been introduced into the comprehensive treatment of glioma. Bevacizumab is one of the VEGF inhibitors and has been reported to relieve the edema caused by glioma (26). But bevacizumab only prolonged progression-free survival (PFS), and the improvement of overall survival (OS) was not been observed. Meanwhile, immunomodulatory therapy has also been widely studied in glioma. Although researchers have reported that PD-1 antibody therapy could not prolong the overall survival of recurrent GBM patients, neoadjuvant PD-1 antibody therapy could stimulate the infiltration of T cells in the tumor microenvironment and induce immune responses to enhance the effect of immunotherapy (27, 28). Therefore, searching for a new target that can influence the glioma immune microenvironment is expected to enhance the effect of immunotherapy.

As a member of the immunoglobulin superfamily, TREM2 can regulate inflammatory response and plays an important role in the process of innate and adaptive immune response (29). An increasing number of studies have shown that TREM-2 is involved in the progression of inflammatory diseases such as pulmonary inflammatory diseases, liver inflammatory diseases, periodontitis, inflammatory bowel disease, sepsis, Alzheimer’s disease, and other inflammatory-related diseases, playing a pivotal role in weakening or aggravating the development of the disease (12, 13, 18, 23). Meanwhile, most studies have shown that TREM2 is a negative regulator of inflammation, which can inhibit the secretion of cytokines, regulate the development and function of dendritic cells, microglia and osteoclasts, regulate phagocytosis, eliminate bacteria, promote anti-inflammatory effects, and protect the body (30, 31). However, some studies have shown that TREM-2 plays a dual role in inflammatory-related diseases, causing adverse effects on the body (32, 33). In summary, more and more evidence show that TREM-2 participates in the regulation of inflammatory response and plays a key role in the immune response.

Our findings have shown that TREM2 expression is related to glioma malignant phenotype. TREM2 is highly expressed in mesenchymal subtype glioma which is characterized by stronger immunosuppression. Moreover, it is highly enriched in the phenotype of known malignant molecules, such as IDH wildtype status and 1p/19q co-deletion status. All these results indicated that TREM2 expression was associated with more malignant biologic processes. Through an in-depth analysis of the biological functions of TREM2 in glioma, we found that TREM2 played an important role in glioma-induced immune suppression. GSVA analysis results revealed that TREM2 suppressed T cell-mediated immune response. A previous study has reported the enhanced expression of immune checkpoints in mesenchymal subtype cancers. Based on this, we performed Pearson correlation analysis and found that TREM2 expression was consistent with the expression of inhibitory immune checkpoints. These results further verified our hypothesis that TREM2 may improve glioma cell immune escape by enhancing the expression of immune checkpoints. By interacting with tumor-infiltrating components, glioma cells evade immune surveillance. CIBERSORT analysis has shown that TREM2 is significantly correlated with M2 macrophages. M2 macrophages are derived from myeloid cells and play a role in tumor support compared with pro-inflammatory M1 macrophages (34, 35). Single-cell sequencing analysis and polychromatic immunohistochemical staining demonstrated that TREM2 was expressed on M2 macrophages and microglia, and TREM2 may remodel the microenvironment *via* macrophages. Furthermore, macrophage polarization experiment and co-culture system have shown that knocking down TREM2 decreased the M2 polarization, and inhibited the co-cultured glioma cells invasiveness. These findings suggested that TREM2 was expressed in M2 macrophages. Meanwhile, TREM2 regulated the M2 phenotype polarization and augmented co-cultured glioma invasiveness.

Importantly, high expression of TREM2 was associated with poor patient prognosis. Univariate and multivariate analyses indicated that high expression of TREM2 predicted lower survival. As a result, TREM2 may serve as a potential prognostic predictor for glioma patients.

Combination therapy will be the mainstream treatment for glioma in the future (36, 37). Neurosurgery, radiotherapy, chemotherapy, targeted therapy, and immunotherapy will be integrated with glioma comprehensive treatment. Our study has proposed a novel potential biomarker TREM2. Inhibition of its expression may enhance the efficacy of immune checkpoint inhibitors and provide a new perspective for immunotherapy.

## 4 Materials and methods

### 4.1 Data collection

All RNA sequencing data and clinical information of patients with diffuse glioma were obtained from three independent data sets, including 699 TCGA-sourced samples (http://cancergenome.nih.gov/), 325 CGGA-sourced samples (http://www.cgga.org.cn) and 693 CGGA2019-sourced samples (http://www.cgga.org.cn).

Molecular pathological results of each glioma patient, including isocitrate dehydrogenase (IDH) mutation and chromosome 1p/19q co-deletion were evaluated in our previous works (38, 39). Paraffin sections of glioma tissue were obtained from the CGGA database. Overall survival of patients was estimated from diagnosis to death or the last follow-up. The single-cell databases GSE70630, GSE89567, and GSE84465 were downloaded from Gene Expression Omnibus (GEO) database. All clinical information of patients was presented in **Table 1**.

Table 1Sample information.

**Table:** 

Characteristics (CGGA)	No. of Patients (n=325)
Age	
<45	191
≥45	134
Gender	
Male	203
Female	122
WHO Grade	
Grade II	103
Grade III	79
Grade IV	139
NA	4
TCGA Subtypes	
Proneural	102
Neural	81
Classical	74
Mesenchymal	68
Radiotherapy+TMZ Chemotherapy	
Yes	154
No	24
Radiotherapy	
Yes	258
No	51
NA	16
TMZ Chemotherapy	
Yes	178
No	124
NA	23
IDH1/2 mutation	
Mutation	175
Wildtype	149
NA	1
1p/19q codeletion	
Codeletion	67
Non-codeletion	250
NA	8
MGMT methylation	
Methylation	130
Unmethylation	112
NA	64

**Table:** 

Characteristics (CGGA(2019))	No. of Patients (n=693)
Age	
<45	382
≥45	310
NA	1
Gender	
Male	398
Female	295
WHO Grade	
Grade II	188
Grade III	255
Grade IV	249
NA	1
TCGA Subtypes	
Proneural	296
Neural	167
Classical	83
Mesenchymal	147
Radiotherapy+TMZ Chemotherapy	
Yes	413
No	67
Radiotherapy	
Yes	509
No	113
NA	71
TMZ Chemotherapy	
Yes	457
No	151
NA	85
IDH1/2 mutation	
Mutation	356
Wildtype	286
NA	51
1p/19q codeletion	
Codeletion	145
Non-codeletion	478
NA	70
MGMT methylation	
Methylation	127
Unmethylation	73
NA	492

**Table:** 

Characteristics (TCGA)	No. of Patients (n=699)
Age	
<45	296
≥45	340
NA	63
Gender	
Male	368
Female	268
NA	63
*WHO Grade*	
Grade II	223
Grade III	245
Grade IV	168
NA	63
TCGA Subtypes	
Proneural	250
Neural	115
Classical	92
Mesenchymal	105
NA	137
IDH1/2 mutation	
Mutation	443
Wildtype	246
NA	10
1p/19q codeletion	
Codeletion	172
Non-codeletion	520
NA	7
MGMT methylation	
Methylation	492
Unmethylation	168
NA	39

Number of glioma patients engaged in our study was listed. All patients were stratified with age, clinicopathological characteristics and treatment options respectively.

### 4.2 Receiver operator characteristic curve

The expression of TREM2 and glioma transcriptome subtype was used for ROC analysis. We used the pROC package of R software to draw the sensitive-specificity curve. Area Under Curve (AUC) represented the accuracy of TREM2 to predict the mesenchymal gliomas (40).

### 4.3 Gene ontology and kyoto encyclopedia of genes and genomes analysis

First, Pearson correlation analysis was performed to generate a gene list that was most related to TREM2. Then, the gene list was uploaded to the DAVID bioinformatics resource (version 6.7) to explore the biological functions and related signaling pathways (41). Finally, GO results of this gene list were visualized by heat map after Spearman correlation analysis.

### 4.4 Gene set variation analysis

We have described this method in our previous study (42). We investigated all identified biological functions to find out the most relevant type to TREM2.

### 4.5 CIBERSORT

To estimate the relative abundance of tumor-infiltrating immune cells in tumor mass from CGGA and TCGA databases, we used a reference set with 22 identified signature gene profiles of immune cell subtypes by the online analysis platform CIBERSORT (https://cibersort.stanford.edu) (43).

### 4.6 Uniform manifold approximation and projection analysis

UMAP analysis estimated a topology of the high-dimensional data and uses this information to construct a low-dimensional representation that preserves relationships present in the data (44). Firstly, the R package Seurat was carried out to cluster cells in different grades of glioma patients’ databases. Meanwhile, the batch effect was removed before the clustering. After normalizing the single-cell data, UMAP analysis was performed to reduce the dimensionality of cells with default parameters. Finally, cluster biomarkers were used to identify each cell group.

### 4.7 Kaplan-Meier plotter

We analyzed the prognostic value of TREM2 expression level in all grades of glioma by using the Kaplan-Meier survival curve and Cox regression analysis in two databases. We computed the log-rank p-value and HR with 95% confidence intervals.

### 4.8 Immunohistochemical staining

Five-micrometer-thick sections were placed in a 65°C oven to deparaffinized for 3 hours. Then the samples were dewaxed in xylene, rehydrated in graded ethanol, and rinsed in distilled water sequentially. Subsequently, antigen retrieval was performed with Tris-EDTA buffer (PH=9.0) and treated with hydrogen peroxide for 10 minutes. After blocking 1 hour, the tissue samples were incubated with primary TREM2 (CST, 91068S, 1:200) antibody at 4°C overnight. Then, the samples were rinsed, and incubated with HRP goat-rabbit IgG H&L (Abcam, ab97051) secondary antibody at 1/500. The samples were counter-stained with hematoxylin and photographed with an optical microscope.

### 4.9 Opal polychromatic immunohistochemical staining

Samples preparation and processing were the same as those of normal immunohistochemical staining. Each stain cycle began with blocking. Primary antibodies were CD3 (Abcam, ab16669, 1:100), SOX2 (Abcam, ab93689, 1:100), CD163 (Abcam, ab156769, 1:100), TMEM119 (Abcam, ab209064, 1:200) and TREM2 (CST, 91068S, 1:200) in sequence. Secondary antibodies were Opal HRP Polymer MS+Rb. Fluorescence-enhanced dyes were Opal 520, Opal 540, Opal 570, Opal 620, and Opal 650. Vectra 3 software was used to generate images.

### 4.10 Cell culture and macrophages polarization

THP-1 cells were cultured in RPMI1640 media supplemented with L-glutamine, 1% penicillin and streptomycin, β-mercaptoethanol and 10% fetal bovine serum at 37°C under a humidified, 5% CO2 atmosphere. THP-1 cells were differentiated to M0 macrophages by successively treated with 25 nM phorbol 12-myristate 13-acetate (PMA) for 48h, washed and incubated with normal RPMI1640 media for 24h, and then incubated with recombinant human GM-CSF for 96h. For M2 polarization, 50% of the complete RPMI1640 medium was added and incubated for 48 h. Then the M2 macrophages were obtained by removing the culture medium and culturing cells for an additional 48h in the M2 medium with recombinant human M-CSF (5, 34). Meanwhile, 10nmol si-RNA-TREM2 was added to the control group for 48h and then replaced with normal RPMI1640 media for another 24h. Then the M2 macrophage polarization process was repeated. Co-culture System: The THP-1 derived M2 macrophages were generated in a 0.4 mm transwell insert for 6-well plate (2*10^5^ cells/well) and washed with fresh DMEM media. The glioma cells were co-cultured with differentiated THP-1 cells for 5 days and were harvested for subsequent assays.

### 4.11 Transwell invasion assay

The transwell invasion assay was performed in 24-well plates with a 6.5mm insert transwell chamber with 8μm polycarbonate membrane (Corning) pre-coated Matrigel (Corning). The single cell suspension was added into to upper chamber with 5*10^4^ cells in 200μl culture medium with 2% FBS, and 500μl culture medium with 20% FBS was added into the lower chamber. After 48 h, discarded the solution in the upper chamber and wiped the upper layer of the membrane. Move the chamber into 4% PFA to fix for 5 min. Stain the membrane with crystal violet for 5 min. Finally, obtained the photographs on the microscope. All experiments were performed in triplicate.

### 4.12 Western blot assay

total protein was extracted after cells transfected with siRNA. After BCA quantification, proteins were denatured by boiling in 5x protein loading buffer. PVDF membrane was employed for transferring following electrophoresis at 80V in Tris-Glycine-SDS buffer. Blocking in 5% skim milk powder at room temperature for two hours. Prepared primary antibody solutions targeting TREM2 (CST, 91068S, 1:1000), CD163 (Abcam, ab156769,1:1000), and GAPDH (Proteintech, 60004-1-Ig, 1:2000) and incubated overnight at 4 degrees Celsius. The next day, wash three times for 10 minutes with TBST buffer. Incubate HRP-conjugated Affinipure Goat Anti-Mouse/Rabbit IgG (H+L) (Proteintech, 1:4000) for 1 hour at room temperature and wash 3 times with TBST buffer for 10 minutes each time. Chemical imaging was performed with a Bio-Rad imaging system. Image was processed using Image Lab (Version 6.0.1).

### 4.13 Immunofluorescence staining

Cells were washed with PBS several times and then fixed with 4% paraformaldehyde for another 10min. Then 0.3% Triton was added to confocal dishes for 15min. After blocking with 5% BSA for 1h, primary antibodies were added to the dishes (TREM2, CST, 91068S, 1:200; CD163, Abcam, ab156769, 1:100) and incubated overnight at 4°C. Secondary antibodies (DyLight 488 goat anti-rabbit polyclonal antibody, Abcam, ab96899, 1:200; DyLight 594 goat anti-mouse polyclonal antibody, Abcam, ab96881, 1:200) were used for 1h at ambient temperature. The cells were washed three times. Finally, Prolong™ Diamond Antifade Mountant with DAPI (Invitrogen, P36962) was added to the dish, and images were taken with confocal microscopy.

### 4.14 Statistical analysis

All statistical analysis and figures were performed with R packages, including ggplot2, pheatmap, corrgram, circlize, and survival, in the statistical software environment R (version 3.5.1). Data collected from at least three independent experiments were expressed as mean ± standard deviation. P < 0.05 was considered statistically significant. The prognostic value of TREM2 was estimated by univariate and multivariate Cox models using SPSS statistical software (version 25.0).

## Data availability statement

The datasets presented in this study can be found in online repositories. The names of the repository/repositories and accession number(s) can be found within the article/**Supplementary Materials**.

## Ethics statement

Written informed consent was obtained from the individual(s) for the publication of any potentially identifiable images or data included in this article.

## Author contributions

MY, YC: data analysis, laboratory work and manuscript writing. YZ, BP: data collection and organization of CGGA database. PW: data collection and organization of TCGA database. GL, TJ, FZ: conception, supervision, and design of the manuscript. All authors contributed to the article and approved the submitted version.
