# Supplementary material for: TREM2 is associated with tumor immunity and implies poor prognosis in glioma
Source: Front Immunol. 2023 Jan 11;13:1089266. doi: 10.3389/fimmu.2022.1089266 (PMC9874686; doi:10.3389/fimmu.2022.1089266)
Supplement: Supplementary file 1 [file DataSheet_1.docx]

Supplementary Material

## Supplementary Figures


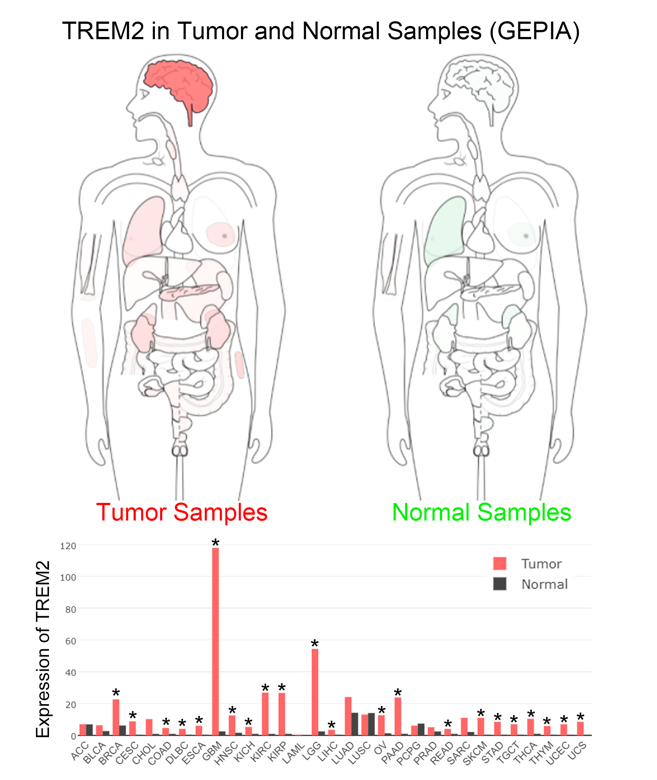


**Supplementary Figure 1**. TREM2 was highly expressed in glioma patients. TREM2 showed a significantly higher expression in GBM (WHO grade IV) compared with normal brain tissue in GEPIA online analysis.


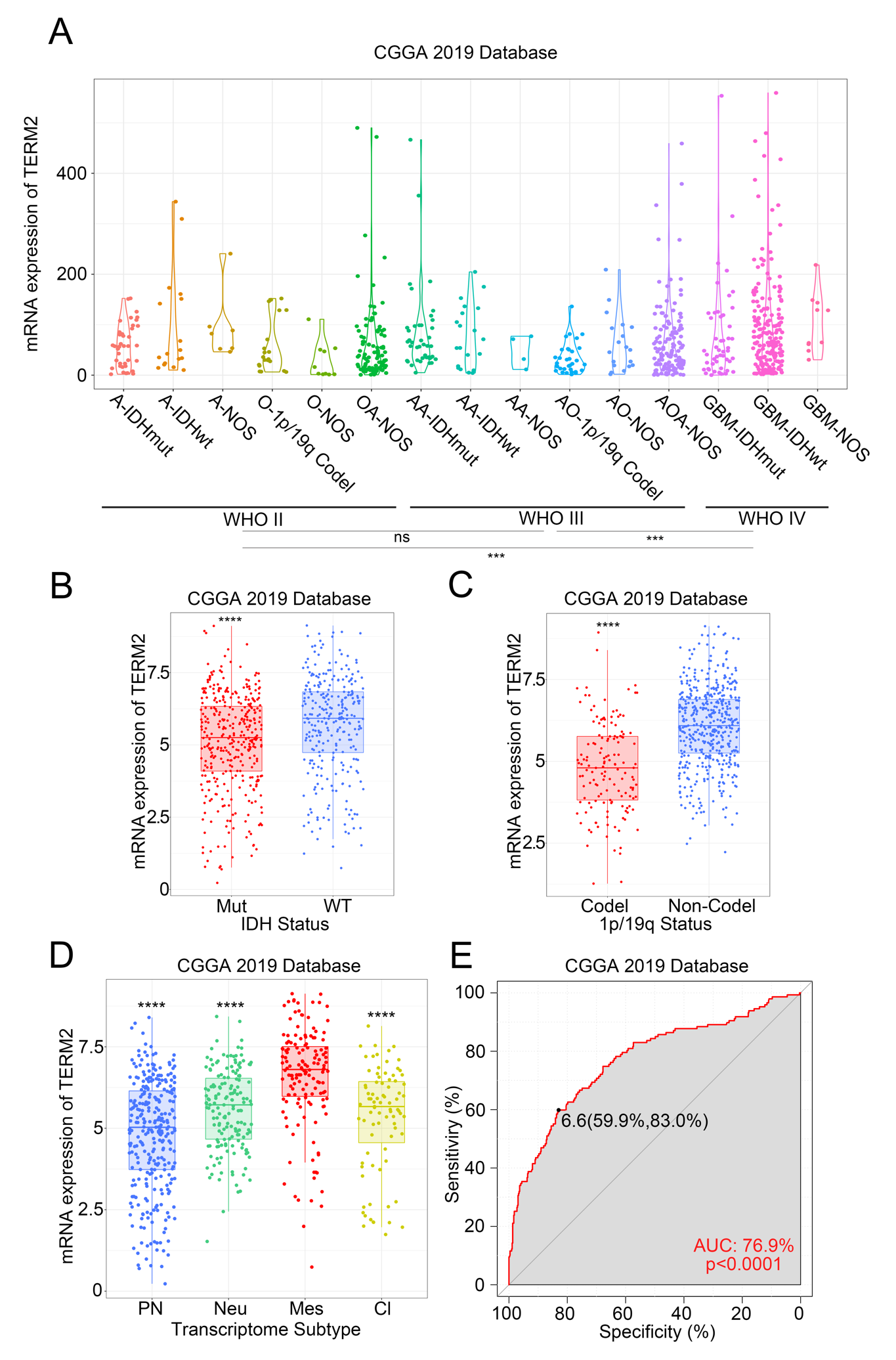


**Supplementary Figure 2**. High expression of TREM2 was associated with malignant glioma. (A) TREM2 was significantly increased in GBM (WHO grade IV) in the CGGA2019 database. (B) TREM2 was significantly increased in IDH wildtype gliomas in the CGGA2019 database (Mut: IDH mutation; WT: IDH wildtype). (C) TREM2 was significantly increased in 1p/19q non-codeletion gliomas in CGGA2019 databases (Codel: 1p/19q codeletion; Non-Codel: 1p/19q non-codeletion). (D) TREM2 was highly expressed in the Mesenchymal subtype in the CGGA2019 database. (E) ROC curve analysis showed that TREM2 was high sensitivity and specificity to predict the Mesenchymal subtype in the CGGA2019 database. A: Astrocytoma, O: Oligodendroglioma, OA: Oligoastrocytoma, AA: Anaplastic astrocytoma, AO: Anaplastic oligodendroglioma, AOA: Anaplastic Oligoastrocytoma, GBM: Glioblastoma. ns, **, *** and **** indicate no statistical difference, p < 0.01, p<0.001 and p < 0.0001, respectively.


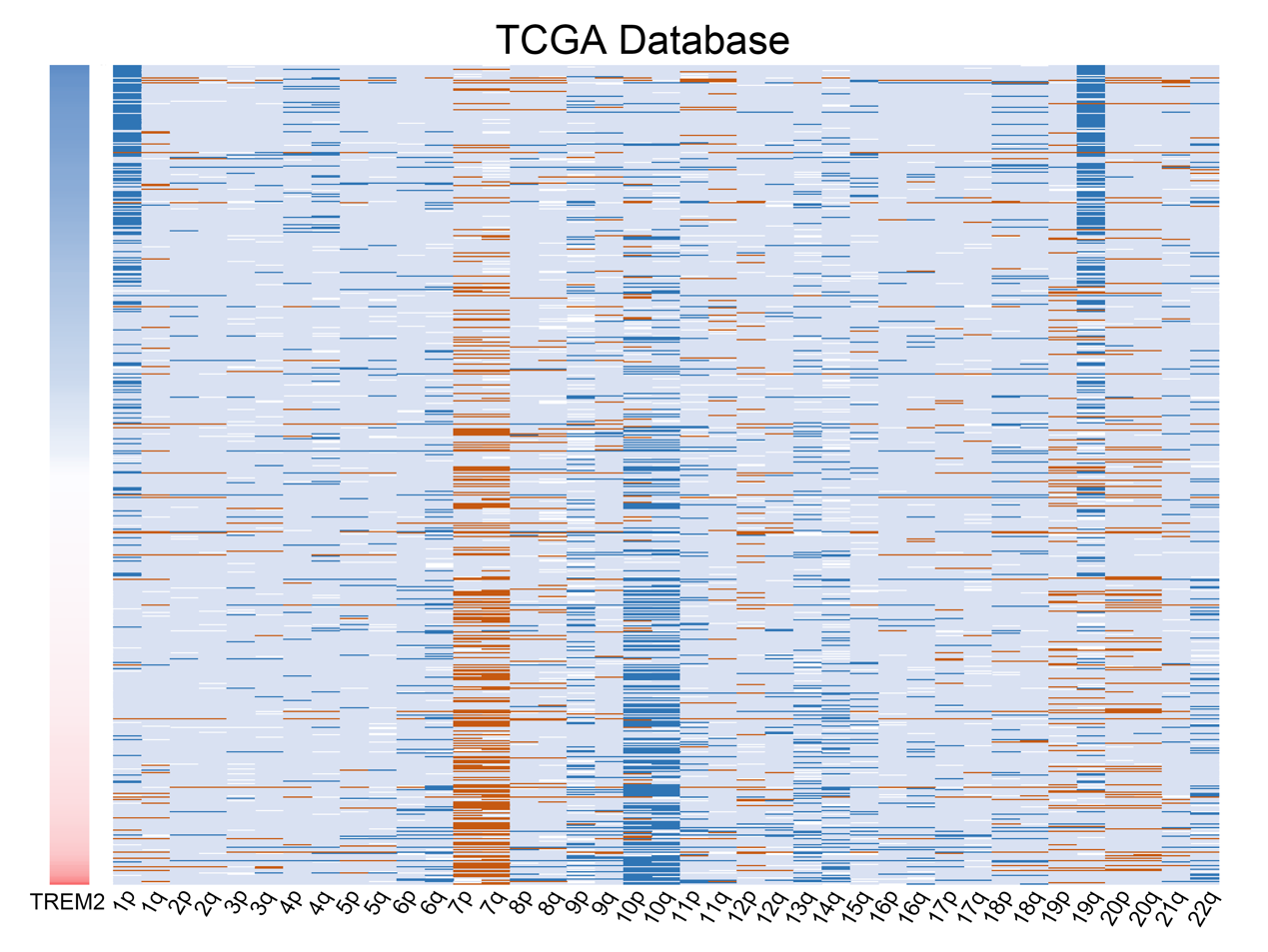


**Supplementary Figure 3**. TREM2 was associated with chromosome instability in glioma. The heatmap of TREM2 expression and chromosome status in TCGA database. The red box represents chromosome amplification and the blue box represents chromosome deletion.


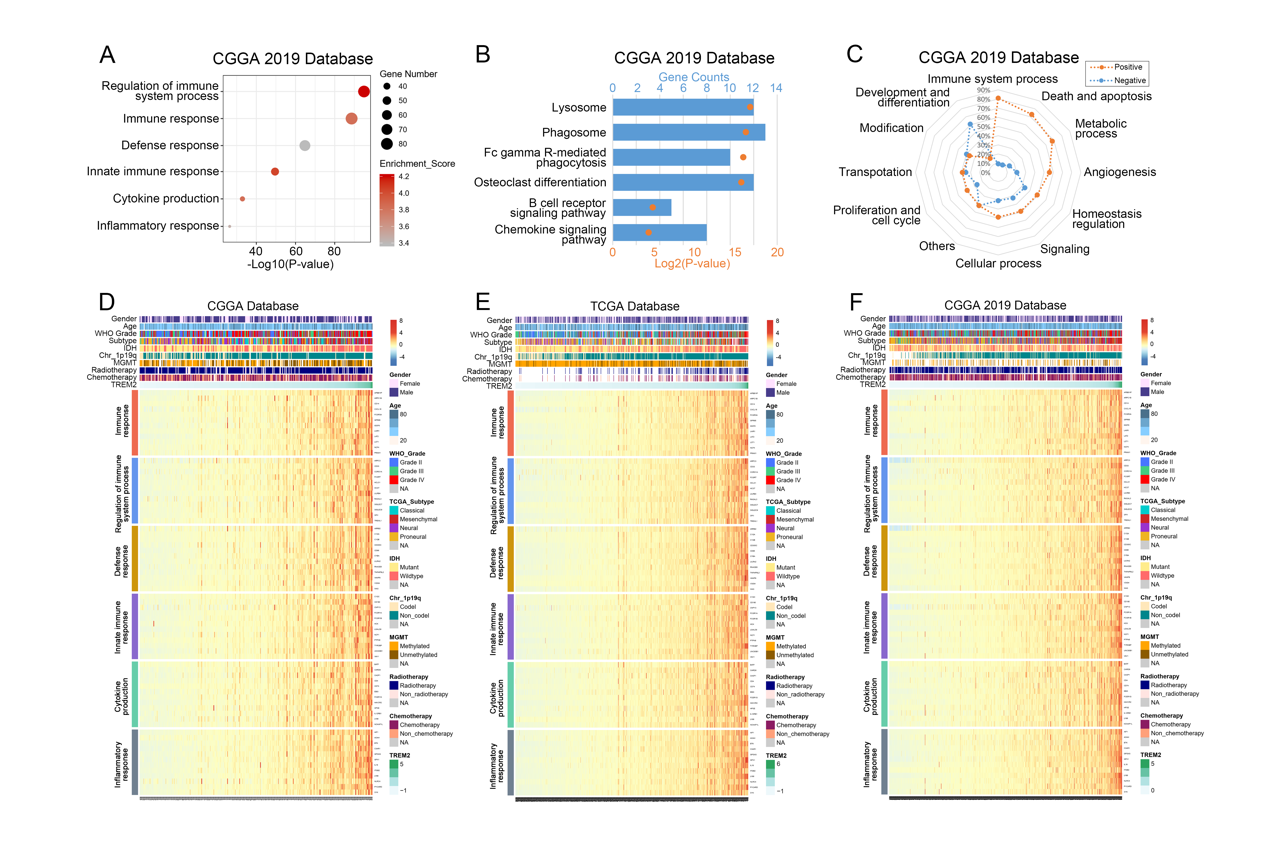


**Supplementary Figure 4**. TREM2 was closely related to immune functions in glioma. (A) Gene ontology analysis showed that TREM2 was mostly associated with immune response, regulation of immune system process, defense response, and inflammatory response in the CGGA2019 database. (B) KEGG pathway analysis showed that TREM2 was mostly involved in the immune response-related pathway in both CGGA2019 databases. (C) TREM2 had a positive correlation with 80.87% of biological functions of the immune system process in the CGGA2019 database. (D-F) Most immune process-related genes were significantly and positively correlated with TREM2 expression in both CGGA, TCGA, and CGGA2019 databases.


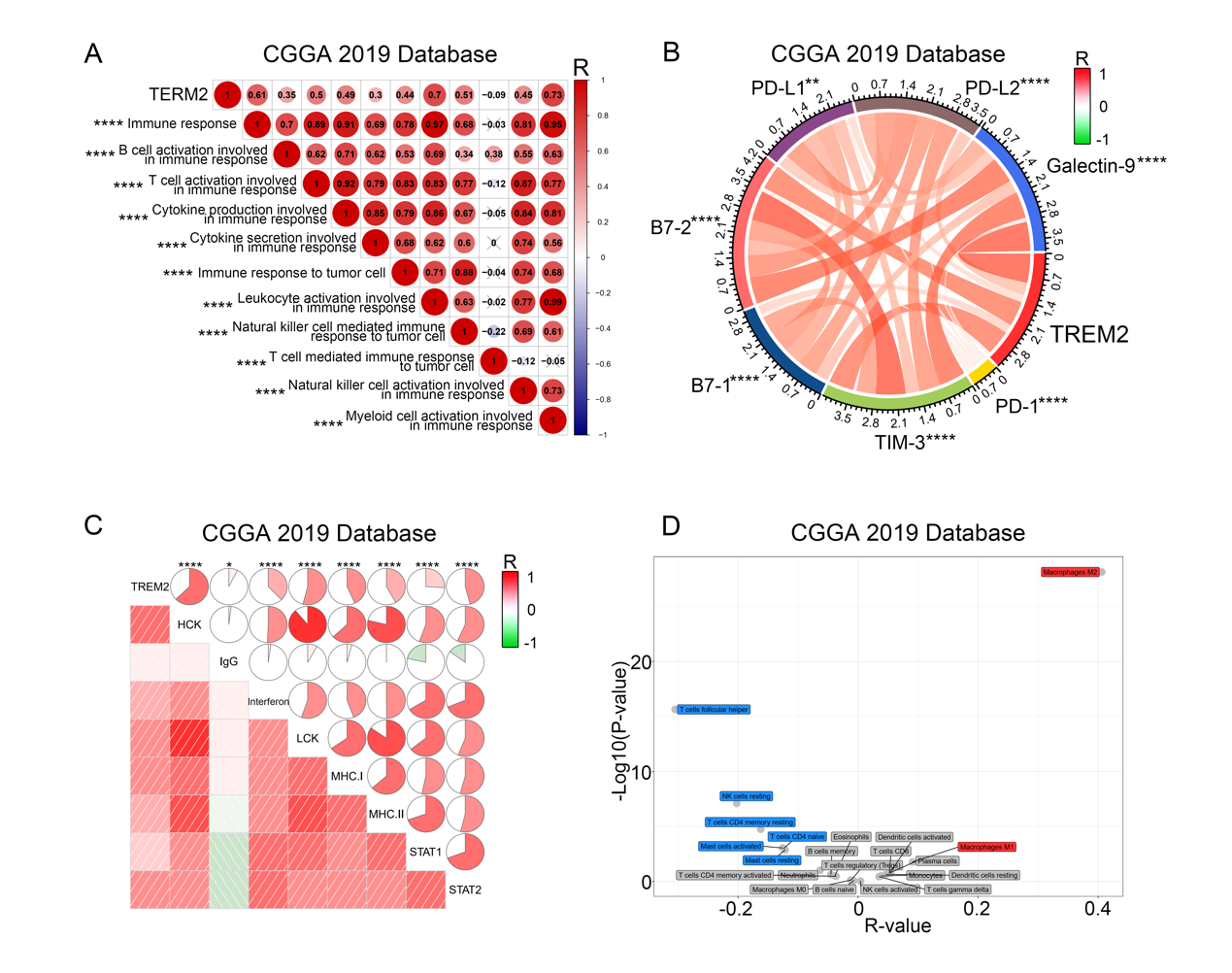


**Supplementary Figure 5**. TREM2-related immune process in CGGA2019 database. (A) The correlation coefficient between TREM2 and immune function scores. (B) The Pearson correlation coefficient between TREM2 and immune checkpoint expression. (C) The relationship between TREM2 and inflammatory activities. The correlation between TREM2 and other functions was analyzed by Pearson correlation analysis. (D) The relationship between TREM2 and infiltrating immune cells. *, ** and **** indicates p<0.05, p<0.01 and p < 0.0001, respectively.


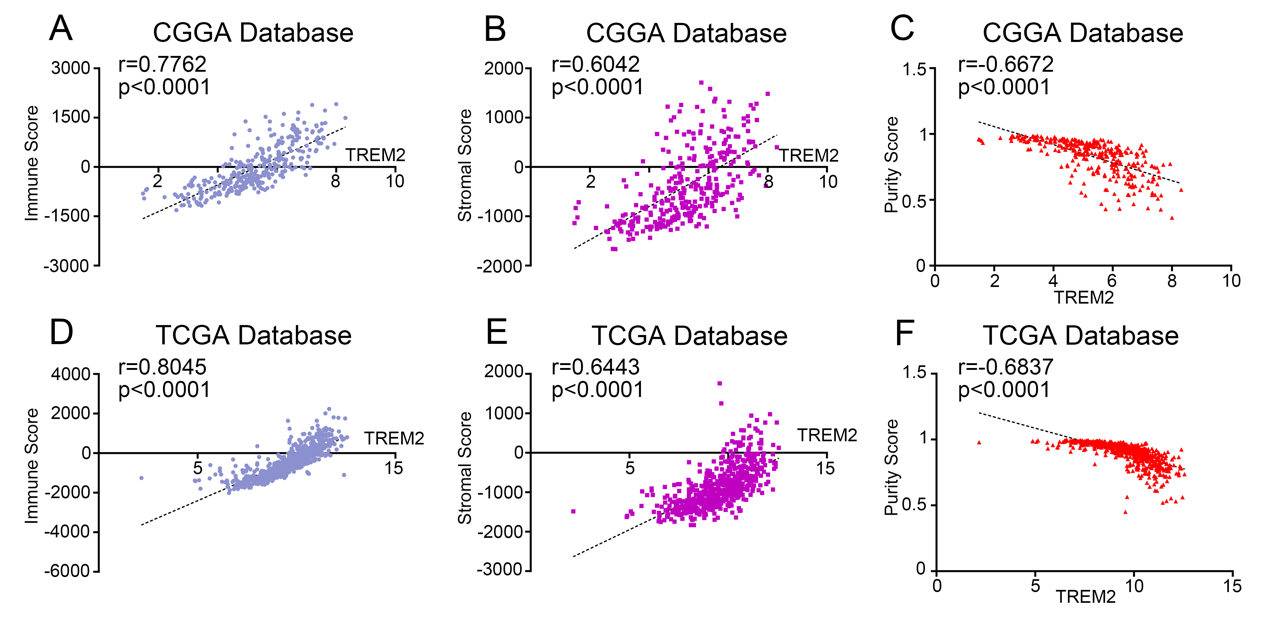


**Supplementary Figure 6**. TREM2 revealed more infiltrated immune cells in both CGGA and TCGA databases. (A, D) The scatterplot showed a significant positive correlation between immune score and TREM2 expression. (B, E) A significant positive correlation was found between stromal score and TREM2 expression. (C, F) Calculated tumor purity was negatively correlated with TREM2 expression.
